# No dose-response relationship of clarithromycin utilization on cardiovascular outcomes in patients with stable coronary heart disease: Analysis of Taiwan’s national health insurance claims data

**DOI:** 10.3389/fcvm.2022.1018194

**Published:** 2022-10-26

**Authors:** Ben-Hui Yu, Yen-Chun Chen, Yi-Da Li, Wen-Yen Chiou, Yi-Chun Chen

**Affiliations:** ^1^Department of Radiation Oncology, Dalin Tzu Chi Hospital, Buddhist Tzu Chi Medical Foundation, Chiayi, Taiwan; ^2^Division of Hepato-Gastroenterology, Department of Internal Medicine, Dalin Tzu Chi Hospital, Buddhist Tzu Chi Medical Foundation, Chiayi, Taiwan; ^3^School of Medicine, Tzu Chi University, Hualien, Taiwan; ^4^Division of Cardiology, Department of Internal Medicine, Dalin Tzu Chi Hospital, Buddhist Tzu Chi Medical Foundation, Chiayi, Taiwan; ^5^Division of Nephrology, Department of Internal Medicine, Dalin Tzu Chi Hospital, Buddhist Tzu Chi Medical Foundation, Chiayi, Taiwan

**Keywords:** clarithromycin, dose-response relationship, all-cause mortality, cardiovascular morbidity, cause-specific mortality

## Abstract

**Background:**

Clarithromycin is widely used to treat various bacterial infections and has been reported to have potential cardiovascular risk. However, it is uncertain whether this association was dose dependent and confounded by indication bias in patients with stable coronary heart disease (CHD).

**Methods:**

This cohort study retrospectively analyzed a national health insurance claims data from Taiwan’s 2005 Longitudinal Generation Tracking Database. We used a new-user design and 1:1 propensity score matching. A total of 9,631 eligible clarithromycin users and 9,631 non-users in 2004–2015 were subject to final analysis. All patients were followed-up after receiving clarithromycin or on the matched corresponding date until occurrence of cardiovascular morbidity in the presence of competing mortality, all-cause and cause-specific mortality, or through the end of 2015. The effect of cumulative dose, exposure duration, and indications of clarithromycin on cardiovascular outcomes were also addressed.

**Results:**

Clarithromycin use, compared with non-use, was associated with higher risk for all-cause [adjusted hazard ratios (aHR), 1.43; 95% confidence interval, 1.29–1.58], cardiovascular (1.35; 1.09–1.67), and non-cardiovascular (1.45; 1.29–1.63) mortality, but not for overall cardiovascular morbidity. Further analysis of individual cardiovascular morbidity demonstrated major risk for heart events (1.25; 1.04–1.51) in clarithromycin users than non-users. However, there was no relationship of cumulative dose, exposure duration, and indications of clarithromycin on cardiovascular outcomes. Analyses of the effects over time showed that clarithromycin increased cardiovascular morbidity (1.21; 1.01–1.45), especially heart events (1.39; 1.10–1.45), all-cause (1.57; 1.38–1.80), cardiovascular (1.58; 1.20–2.08), and non-cardiovascular (1.57; 1.35–1.83) mortality during the first 3 years. Thereafter, clarithromycin effect on all outcomes almost dissipated.

**Conclusion:**

Clarithromycin use was associated with increased risk for short-term cardiovascular morbidity (especially, heart events) and mortality without a dose-response relationship in patients with stable CHD, which was not dose dependent and confounded by indications. Hence, patients with stable CHD while receiving clarithromycin should watch for these short-term potential risks.

## Introduction

Cardiovascular disease (CVD) is a growing worldwide burden, with approximately 17 million deaths globally in 2017, and is estimated to be over 23 million in 2030 (1). CVD includes coronary heart disease (CHD), and chronic infection has been advocated as a possible contributing factor to CHD (2). Several infectious agents, such as *Chlamydia pneumoniae*, *Helicobacter pylori*, cytomegalovirus, and herpes simplex virus (3, 4), were associated with the development of atherosclerosis. Besides, inflammation also mediates atherogenesis (5). These findings supported the hypothesis that some microorganisms were related to coronary artery disease. In addition to antiplatelet drugs, previous studies also investigated antibiotics as potential secondary prevention agents (6–10). They evaluated the safety and efficacy of macrolide antibiotics in patients with CHD. In an earlier study (11), *C. pneumoniae* was eradicated from atherosclerotic plaques using roxithromycin (a macrolide antibiotic) so roxithromycin might be helpful for patients with CHD. Therefore, several prospective, randomized, placebo-controlled trials investigated the role of macrolide antibiotics and the risk of cardiovascular events (6–10). However, the efficacy of macrolide antibiotics was conflicting in these studies. It is hard to decide if the macrolide antibiotics were effective or ineffective in CHD as secondary prevention because their dosages, durations, the timing of dispensing, and even the baseline characteristics of treated subjects were not comparable between the above mentioned studies. Besides, all enrolled subjects did not show any indication for antibiotics use such as acute infection in those prospective studies. That is, antibiotics were just used as secondary prevention for CHD. Other observational studies also reported the outcomes of clarithromycin for cardiac events among patients with acute infectious disease (12–16). These observational studies still showed conflicting results.

Different baseline characteristics, such as confounding drugs, might partly account for inconsistent results. When we considered confounding drugs, the use of ivabradine (17) and the dose of aspirin (18) may play crucial roles in CHD patients. Different patients may require different aspirin dosages to achieve complete inhibition of platelet function. A clinical study (18) reported that there was an inter-individual variability in response to the antiplatelet effect of standard doses of aspirin (150, 300 mg/day), the response to aspirin 300 mg/day was enhanced in resistant patients when compared to 150 mg/day, and there was a significant association between aspirin resistance and atherothrombotic risk factors such as diabetes, hyperlipidemia and obesity. In Taiwan, the dose of 100 mg/day of aspirin is recommended, and almost all patients receive aspirin 100 mg/day (19, 20). Hence, we did not consider the impact of aspirin dosage in the present study. A prospective randomized controlled study (17) investigated the effect of ivabradine as add-on treatment on the high sensitivity C-reactive protein (hsCRP) levels in patients with non ST-segment elevation acute coronary syndromes (NSTE-ACS). They found that ivabradine effectively and safely decreased heart rate in NSTE-ACS patients, which was associated with hsCRP reduction. According to the regulation of Taiwan’s National Health Insurance (NHI), ivabradine was reimbursed in 2014 (21). The enrolled population in this study was from 2000 to 2015, so we did not take into consideration.

Clarithromycin is widely used to treat various bacterial infections. Patients with or without a history of cardiovascular disease may receive such treatment if they are indicated. However, the results remained conflicting. These observational studies (12–16) did not exclude the history of macrolide use in their baseline characteristics, which might affect the cardiovascular outcome of subsequent clarithromycin use. Some studies excluded baseline cardiovascular disease (15, 16), so it was uncertain whether their results could apply to patients with a history of heart disease. Most importantly, those studies did not clearly address if these results were confounded by clarithromycin indications. Furthermore, the CLARICOR trial with 3 years’ follow-up (7) demonstrated higher cardiovascular death in patients with stable CHD taking clarithromycin; however, the CLARICOR trial with 10 years’ follow-up (22) demonstrated that clarithromycin increased cardiovascular death during the first 3 years, not the last 4 years, in patients with stable CHD who were not on statin. This paradoxical result implied that the effect of clarithromycin on cardiovascular death might differ between short-term and long-term follow-ups. The durations observed in the most aforementioned studies were equal to or less than three years. Moreover, if clarithromycin harms the heart, the dose-response relationship of clarithromycin utilization with cardiovascular outcomes remains unknown. Hence, further research is warranted to better understanding this association.

Taiwan has had a mandatory-enrollment NHI program since 1995, and a very high universal coverage rate (>99%) by the end of 2010, allowing long-term follow-up studies (23). Due to complete nationwide coverage, we implemented the new-user design and employed propensity score matching and competing risk analysis to reappraise the survival and cardiovascular outcomes in patients with stable CHD with or without clarithromycin administration.

## Materials and methods

### Data source

This nationwide retrospective cohort study was based on a national health insurance claims data from Taiwan’s 2005 Longitudinal Generation Tracking Database (LGTD2005), which includes 2 million de-identified individuals who were randomly sampled in 2005 from all beneficiaries in Taiwan’s NHI program by the Health and Welfare Data Science Center of the Taiwan Ministry of Health and Welfare (24). All medical records between 2000 and 2016 were tracked. Therefore, this study did not require informed consent and was exempted from full review by the Institutional Review Board of the Dalin Tzu Chi Hospital (B10702014 and B11101016), and the LGTD2005 contains comprehensive medical information, except laboratory and lifestyle data, and adopts ICD-9-CM diagnosis codes to define diseases and anatomical therapeutic chemical codes to identify drugs. It was documented that there was no significant difference in age, sex, region, ambulatory care, and inpatient expenditures between the LGTD2005 and NHI programs. Details regarding LGTD2005 and Taiwan’s national health insurance claims data have been reported in our previous work (23, 25–28).

### Identification of study population (stable coronary heart disease cohort)

**FIGURE 1:**
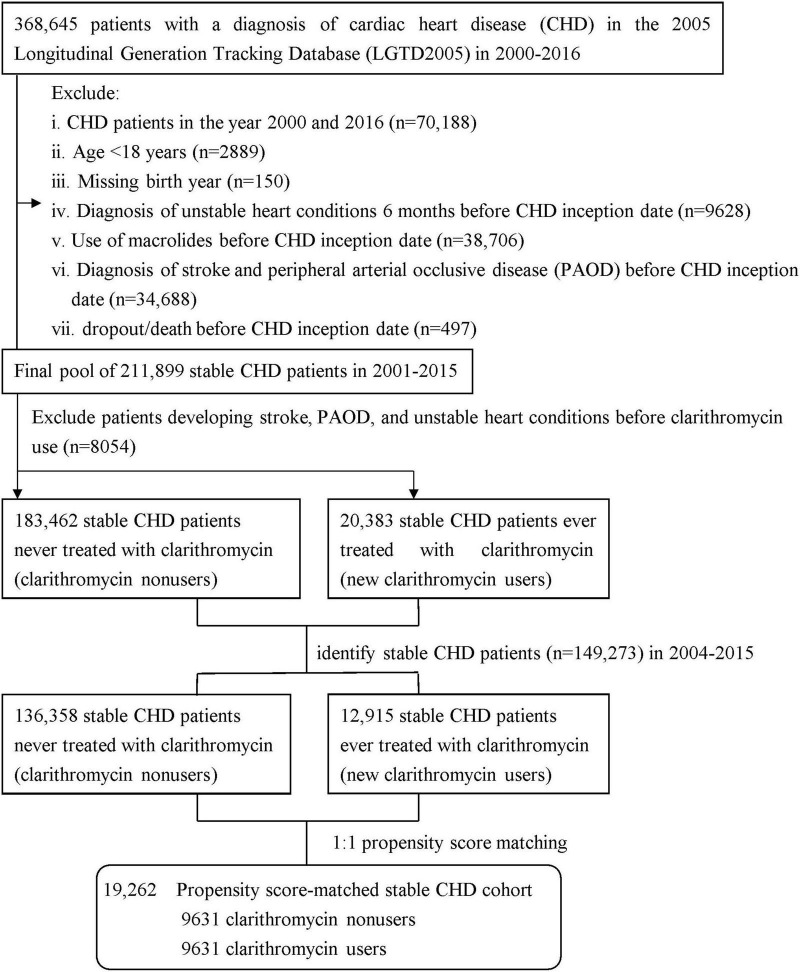
Flowchart of study design.

We identified all patients diagnosed with CHD by ICD-9-CM diagnosis codes (410–414) (29, 30) between January 1, 2000 and December 31, 2016 from the outpatient and inpatient claims (Figure 1). We excluded patients with CHD who were diagnosed in the year 2000 to ensure every patient having 1-year available data prior to their first CHD episode for comorbidity assessment and the year 2016 to avoid misclassification of a clarithromycin user as non-user due to missing data in 2017, aged < 18 years, lacked birth year, ever used macrolides, dropped out/expired, and were diagnosed with stroke and peripheral arterial occlusive disease (PAOD) before CHD inception date, and experienced unstable heart conditions 6 months before CHD inception date. The unstable heart conditions were defined as experience of any of three cardiac interventional procedures, including percutaneous transluminal coronary angioplasty, only cardiac angiography without percutaneous transluminal coronary angioplasty, or cardiac electrophysiological study. Thus, we obtained a final pool of 211,899 patients with stable CHD during 2001–2015. According to the use of clarithromycin, the pool was divided into two groups: clarithromycin users vs. clarithromycin non-users (as controls), the method of which was used in prior research (25, 31, 32). There were 183,462 clarithromycin non-users, namely controls, who were never treated with clarithromycin throughout the study period and 2,383 new clarithromycin users who did not experience unstable heart conditions, stroke, and PAOD before clarithromycin use. To avoid survival bias, we selected eligible patients with CHD from 2004 to 2015: 12,915 clarithromycin users and 136,358 non-users. Each clarithromycin user was propensity-matched with one non-user and, to avoid immortal bias, the baseline for matching was set at the day when clarithromycin commenced in the user group and the corresponding date in the matched non-user group (25, 33). The propensity score was calculated using the logistic regression built on all baseline covariates listed in Table 1 to adjust for the baseline differences between clarithromycin users and non-users. The propensity score model was reliable (Hosmer–Lemeshow test, *p* = 0.52) and provided fair discrimination between two groups (c-index, 0.65). The index date of clarithromycin users was set on the day when clarithromycin administration commenced, and that of non-users was the corresponding day. A total of 19,262 patients with stable CHD were subjected to the final analysis.

**TABLE 1:** Characteristics of study cohort by the use of clarithromycin.

	Propensity score-matched patients with stable CHD (*n* = 19,262)		
	Clarithromycin users	Non-users	
Variable	(*n* = 9,631) *N* (%)	(*n* = 9,631) *N* (%)	*p*-value
Sex, *n* (%)			0.42
Men	4,746 (49.3)	4,802 (49.9)	
Women	4,885 (50.7)	4,829 (50.1)	
Age (year), *n* (%)			0.97
18–49	3,634 (37.7)	3,631 (37.7)	
50–59	2,889 (30.0)	2,912 (30.2)	
60–69	1,810 (18.8)	1,810 (18.8)	
≥ 70	1,298 (13.5)	1,278 (13.3)	
Mean (± *SD*)	53.7 ± 13.8	53.4 ± 14.4	
**Comorbidity, *n* (%)**			
Diabetes	993 (10.3)	983 (10.2)	0.81
Hypertension	3,691 (38.3)	3,679 (38.2)	0.86
COPD	1,338 (13.9)	1,313 (13.6)	0.60
No. of medical visits, *n* (%)			0.83
1–12	3,593 (37.3)	3,632 (37.7)	
13–24	3,145 (32.7)	3,134 (32.5)	
≥ 25	2,893 (30.0)	2,865 (29.8)	
**Confounding drugs use, *n* (%)**			
ACEI/ARB	1,706 (17.7)	1,688 (17.5)	0.73
Aspirin	2,223 (23.1)	2,224 (23.1)	0.99
Statins	638 (6.6)	606 (6.3)	0.35
Ticlopidine	2 (0.02)	1 (0.01)	0.56
Calcium channel blockers	3,295 (34.2)	3,298 (34.2)	0.96
Beta blockers	1,627 (16.9)	1,599 (16.6)	0.59
Diuretics	3,931 (40.8)	3,925 (40.8)	0.93
Antiarrhythmics	2,162 (22.5)	2,153 (22.4)	0.88
Digoxin	48 (0.5)	45 (0.5)	0.76
Nitrates	1,453 (15.1)	1,413 (14.7)	0.42

CHD, coronary heart disease; COPD, chronic obstructive pulmonary disease; ACEI/ARB, angiotensin-converting-enzyme inhibitor/angiotensin II receptor blocker.

### Study outcomes

The primary outcome was all-cause mortality; the second outcome was cardiovascular mortality comprising death from principle diagnoses of CHD, stroke, and PAOD, and non-cardiovascular mortality. We did not take into account the detailed causes of non-cardiovascular mortality during the follow-up. The third outcome was cardiovascular morbidity, including heart events (defined as experience of the above-mentioned unstable heart conditions or having principle diagnosis of CHD), stroke events (defined as having principle diagnosis of stroke), and PAOD events (defined as having principle diagnosis of PAOD). Both cohorts were followed from the index date to the development of study outcomes or the end of the study (December 31, 2015), whichever came first. Death was defined as withdrawal/dropout of the subject from the NHI program (33, 34).

### Covariate assessment

Preexisting comorbidities, including hypertension, diabetes, and chronic obstructive pulmonary disease as a proxy for smoking (35, 36) because smoking can increase the risk for airway infections (37), were identified within 1 year before CHD inception date. The number of medical visits in 1 year before CHD inception date was introduced into multivariate analyses to correct the confounding effect of medical attention, since medical attention may explain some of the remaining risk elevation (23, 26, 38). Ten confounding drugs were considered, including angiotensin-converting enzyme inhibitor/angiotensin II receptor blocker (ACEI/ARB), aspirin, statins, ticlopidine, calcium channel blockers, beta-blockers, nitrates, diuretics, antiarrhythmics, and digoxin.

### Statistical analysis

The baseline differences between clarithromycin users and non-users were compared using *t*-tests for continuous variables and the chi-squared test for categorical variables. Death before cardiovascular morbidity was considered a competing risk event (39). After confirming the assumption of proportional hazards by plotting survival function vs. survival time, and log (-log (survival)) vs. log of survival time, we applied the modified Cox proportional hazard model in the presence of competing risk to examine the association of clarithromycin with cardiovascular morbidity and individual event from heart, stroke, and PAOD, and the Cox proportional hazard model to examine the association of clarithromycin with mortality, with adjustment for all covariates (age per year, sex, comorbidities, number of medical visits, and confounding drugs). We calculated and compared the cumulative incidence of cardiovascular morbidity and individual event from heart, stroke, and PAOD between two groups in data with competing risk using the modified Kaplan-Meier method and Gray’s method (40). We further analyzed the risk of unstable heart conditions, which were a part of heart events and defined as experience of any of the above-mentioned three cardiac interventional procedures, whichever came first. In multivariable stratified analyses, we evaluated the adjusted hazard ratios (aHRs) of all study outcomes for the first 3 years, 3–6 years, and after 6 years of follow-up in relation to clarithromycin use compared to non-use. To assess the dose-response relationship of clarithromycin utilization with the risk of cardiovascular mortality and morbidity, we calculated each patient’s cumulative defined daily dose (cDDD) of clarithromycin according to the WHO’s recommendation (41), the method of which has been used in several retrospective NHI-based research investigating the dose response relationship (38, 42, 43). The DDD metric provides a fixed unit of measurement to allow data from drug utilization studies to be standardized across countries and settings that are independent of price, currencies, pack size and strength, clinical indications, administration route, and treatment purposes, and to enable trends in drug utilization to be assessed and compared between population group (44).

We estimated their mode cDDDs by two (< 3500 *vs.* ≥ 3500) and four (< 2250, 2250–3500, 3500–3750, *vs.* ≥ 37500) levels and mean cDDDs by two levels (<4380 *vs.* ≥ 4380). We also evaluated the risk of cardiovascular mortality and morbidity associated with short-term (≤7 days), medium-term (8–14 days), and long-term (≥15 days) dosing period of clarithromycin prescription. To assess the reliability of our main findings, we conducted several sensitivity analyses. First, we added three comorbidities (hyperlipidemia, renal diseases, and arrhythmias), which were associated with cardiovascular risk. Second, we considered one confounding drug quinolone, which had the potential for cardiovascular risk (45). Third, we used amoxicillin users as controls (16). Fourth, we further addressed if cardiovascular outcomes were related to clarithromycin indications. We performed two subgroups analyses of cardiovascular outcomes by clarithromycin indications for acute respiratory infection, genitourinary tract infection, *Helicobacter pylori* infection, and mycobacterial (tuberculosis and non-tuberculosis) infections, as well as by dosing period of clarithromycin indications for acute respiratory tract infection and *H. pylori* infection. All data were analyzed using SAS (version 9.4; SAS Institute, Inc., Cary, N.C.). A two-sided *p*-value less than 0.05 was considered statistically significant.

## Results

### Patients’ characteristics

In the overall CHD population (*n* = 368,645) in 2000–2016, male gender was significantly higher than female gender (*p* < 0.0001), mean age was 59 years, and the percentage of preexisting diabetes was 19% (data not shown). Among 12,915 clarithromycin users, 9,631 were propensity score-matched to a control set of 9,631 non-users at a 1:1 ratio (Figure 1). Given the type I error α of 0.05, the event rate per year of 0.016 for nonuser group, median follow-up of 4 years, censoring rate of 0.5, and user-to-nonuser ratio of 1:1, it takes 7,100 in both user and nonuser groups to have a power (1-β) of 0.95 to detect a 40% change in hazard ratio. Our sample size of 9631 in each group with aHR of 1.43 suggests a test power greater than 0.95. The average age of both groups was 53 years (Table 1). The proportions of medical visits, patients with diabetes, hypertension, and chronic obstructive pulmonary disease, and those receiving confounding drugs, were similar within both groups.

### Eleven years of patients’ follow-up

The clarithromycin users and non-users were followed up for a mean duration of 4 years (Table 2). The 11-year cumulative incidence of all-cause and non-cardiovascular mortality was significantly higher in the clarithromycin users than non-users, with 16.7% [95% confidence interval (CI), 15.4–18.1%] vs. 14.8% (13.5–16.3%) and 13.6% (12.3–14.9%) *vs.* 11.4% (10.2–12.7%), respectively (*p* < 0.0001). The incidences of cardiovascular mortality and morbidity did not differ between both groups.

**TABLE 2:** Outcomes by the use of clarithromycin.

	Clarithromycin users (*n* = 9,631)	Non-users (*n* = 9,631)	*p*-value	Adjusted HR (95% CI)	*p*-value
All-cause mortality				1.43 (1.29–1.58)*	<0.0001
Mean follow-up (± *SD*)	4.2 ± 2.9	4.2 ± 2.9			
Event (*n*, %)	858 (8.9)	671 (7.0)	<0.0001		
Cumulative incidence (%, 95% CI)	16.7 (15.4–18.1)	14.8 (13.5–16.3)	<0.0001		
Cardiovascular mortality				1.35 (1.09–1.67)*	0.005
Mean follow-up (± *SD*)	4.2 ± 2.9	4.2 ± 2.9			
Event (*n*, %)	190 (2.0)	162 (1.7)	0.13		
Cumulative incidence (%, 95% CI)	3.6 (3.0–4.3)	3.9 (3.1–4.7)	0.10		
Non-cardiovascular mortality				1.45 (1.29–1.63)*	<0.0001
Mean follow-up (± *SD*)	4.2 ± 2.9	4.2 ± 2.9			
Event (*n*, %)	668 (6.9)	509 (5.3)	<0.0001		
Cumulative incidence (%, 95% CI)	13.6 (12.3–14.9)	11.4 (10.2–12.7)	<0.0001		
Cardiovascular morbidity				1.09 (0.94–1.26)^#^	0.26
Mean follow-up (± *SD*)	4.0 ± 2.9	4.1 ± 2.9			
Event (*n*, %)	404 (4.2)	344 (3.8)	0.12		
Cumulative incidence (%, 95% CI)	7.5 (6.6–8.5)	7.1 (6.2–8.1)	0.10		

*SD*, standard deviation; HR, hazard ratio; CI, confidence interval. *Adjusted for all covariates (age per year, sex, comorbidity, number of medical visits, and confounding drugs). ^#^Adjusted for all covariates (age per year, sex, comorbidity, number of medical visits, and confounding drugs) and competing mortality.

### Multivariable-adjusted association of clarithromycin with study outcomes

In multivariable Cox regression analysis (Table 2), clarithromycin use was significantly associated with increased risk for all-cause (aHR, 1.43; 95% CI, 1.29–1.58, *p* < 0.0001), cardiovascular (1.35; 1.09–1.67, *p* = 0.005), and non-cardiovascular (1.45; 1.29–1.63, *p* < 0.0001) mortality, not cardiovascular morbidity (1.09; 0.94–1.26, *p* = 0.26) in the presence of competing mortality.

### Association of clarithromycin with individual event of cardiovascular morbidity

Clarithromycin use, compared with non-use, was significantly associated with increased cumulative incidence (4.7%; 95% CI, 4.1–5.5% *vs.* 4.0%; 3.3–4.7%) and risk for heart events (aHR, 1.25; 95% CI, 1.04–1.51, *p* = 0.018) (Table 3). However, there was no significant difference of stroke and PAOD events between clarithromycin users and non-users.

**TABLE 3:** Individual event of cardiovascular morbidity associated with clarithromycin use.

	Clarithromycin users (*n* = 9,631)	Non-users (*n* = 9,631)	*p*-value	Adjusted HR^#^ (95% CI)	*p*-value
Heart events^†^				1.25 (1.04–1.51)	0.018
Mean follow-up (± *SD*)	4.1 ± 2.9	4.0 ± 2.9			
Event (*n*, %)	271 (2.8)	198 (2.2)	0.004		
Cumulative incidence (%, 95% CI)	4.7 (4.1–5.5)	4.0 (3.3–4.7)	0.003		
Stroke events				0.90 (0.70–1.15)	0.39
Mean follow-up (± *SD*)	4.1 ± 2.9	4.0 ± 2.9			
Event (*n*, %)	127 (1.3)	135 (1.5)	0.37		
Cumulative incidence (%, 95% CI)	2.6 (2.1–3.3)	2.9 (2.3–3.5)	0.40		
PAOD events				0.51 (0.19–1.39)	0.19
Mean follow-up (± *SD*)	4.1 ± 2.9	4.0 ± 2.9			
Event (*n*, %)	6 (0.1)	11 (0.1)	0.19		
Cumulative incidence (%, 95% CI)	0.2 (0.1–0.4)	0.3 (0.1–0.6)	0.19		

*SD*, standard deviation; HR, hazard ratio; CI, confidence interval; PAOD, peripheral arterial occlusion disease. ^†^Defined as experience of unstable heart conditions (defined in Supplementary Table 1) or having a principle diagnosis of CHD, whichever came first. ^#^Adjusted for all covariates (age per year, sex, comorbidity, number of medical visits, and confounding drugs) and competing mortality.

### Association of clarithromycin with unstable heart conditions experiencing any of three cardiac interventional procedures

Only cardiac angiography without percutaneous transluminal coronary, not percutaneous transluminal coronary angioplasty and cardiac electrophysiological study, significantly accounted for increased cumulative incidence (4.1%; 95% CI, 3.5–4.8% vs. 3.2%; 2.6–3.8%) and risk (aHR, 1.32; 95% CI, 1.08–1.61, *p* = 0.007) for unstable heart conditions in clarithromycin users, compared with non-users (Supplementary Table 1).

### Association of cumulative defined daily dose of clarithromycin with cardiovascular outcomes

We estimated mode cDDD by two and four levels and mean cDDD by two levels of clarithromycin and found no dose-response relationship between clarithromycin and cardiovascular outcomes (Table 4).

**TABLE 4:** Association of several approaches to cumulative define daily dose (cDDD) of clarithromycin with cardiovascular outcomes.

Take non-users as the reference	Cardiovascular mortality events,%	Adjusted HR* (95% CI)	*p*-value	Cardiovascular morbidity events,%	Adjusted HR^#^ (95% CI)	*p*-value
**Mode cDDD of clarithromycin by two levels**						
< 3,500	2.4%	2.18 (1.51–3.15)	<0.0001	3.1%	0.95 (0.72–1.25)	0.70
≥ 3,500	1.8%	1.04 (0.80–1.35)	0.77	4.8%	1.18 (1.00–1.40)	0.053
**Mode cDDD of clarithromycin by four levels**						
< 2,250	2.5%	1.90 (1.25–2.87)	0.0025	3.2%	1.01 (0.73–1.39)	0.97
2,250–3,500	2.2%	3.17 (1.38–7.30)	0.007	2.7%	0.78 (0.46–1.35)	0.38
3,500–3,750	1.5%	1.06 (0.75–1.52)	0.73	5.2%	1.30 (1.05–1.61)	0.02
≥ 3,750	2.2%	1.03 (0.70–1.52)	0.88	4.3%	0.99 (0.74–1.31)	0.93
**Mean cDDD of clarithromycin**						
< 4,380	2.0%	1.50 (1.18–1.91)	0.001	4.2%	1.15 (0.97–1.36)	0.10
≥ 4,380	1.9%	0.97 (0.63–1.50)	0.90	4.4%	1.00 (0.74–1.35)	0.99

HR, hazard ratio; CI, confidence interval. *Adjusted for all covariates (age per year, sex, comorbidity, urbanization level, geographic region, enrollee category, number of medical visits, and confounding drugs). ^#^Adjusted for all covariates (age per year, sex, comorbidity, urbanization level, geographic region, enrollee category, number of medical visits, and confounding drugs) and competing mortality.

### Association of dosing period of clarithromycin with cardiovascular outcomes

Short-term (≤7 days), not medium-term (8–14 days) and long-term (≥15 days), dosing period of clarithromycin use, compared with non-sue, was significantly associated with increased risk of cardiovascular mortality (aHR, 1.59; 95% CI, 1.22–2.06) and morbidity (aHR, 1.48; 95% CI, 1.33–1.64) (Table 5).

**TABLE 5:** Association of dosing period of clarithromycin with cardiovascular outcomes.

Clarithromycin prescription	Cardiovascular mortality aHR* (95% CI)	Cardiovascular morbidity aHR^#^ (95% CI)
None (*n* = 9,631)	1 (reference)	1 (reference)
≤7 days (*n* = 6,359)	1.59 (1.22–2.06)	1.48 (1.33–1.64)
8–14 days (*n* = 1,987)	1.04 (0.66–1.64)	1.14 (0.93–1.39)
≥15 days (*n* = 1,285)	0.80 (0.44–1.47)	0.92 (0.72–1.18)

SD, standard deviation; aHR, adjusted hazard ratio; CI, confidence interval. *Adjusted for all covariates (age per year, sex, comorbidity, number of medical visits, and confounding drugs).

^#^Adjusted for all covariates (age per year, sex, comorbidity, number of medical visits, and confounding drugs) and competing mortality.

### Stratified analyses of the effects of clarithromycin over time

**FIGURE 2:**
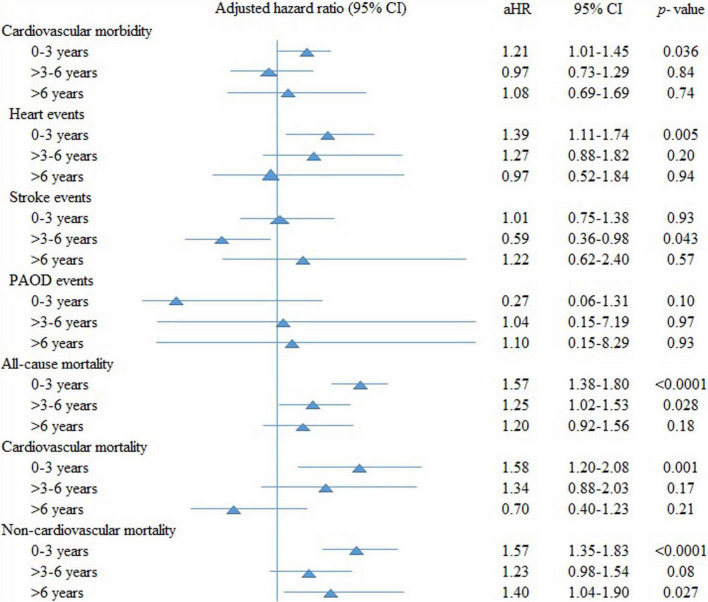
The adjusted hazard ratios (aHRs) of all outcomes stratified by follow-up periods of clarithromycin use compared with non-use. CI, confidence interval; PAOD, peripheral arterial occlusion disease.

Figure 2 examines the aHRs of all outcomes stratified by follow-up periods for ≤3 years, > 3–6 years, and > 6 years of clarithromycin use compared with non-use. Clarithromycin, compared with non-use, increased cardiovascular morbidity (1.21; 95% CI, 1.01–1.45, *p* = 0.036), especially heart events (1.39; 1.11–1.74, *p* = 0.005) rather than stroke and PAOD events, all-cause (1.57; 1.38–1.80, *p* < 0.0001), cardiovascular (1.58; 1.20–2.08, *p* = 0.001), and non-cardiovascular (1.57; 1.35–1.83, *p* < 0.0001) mortality during the first 3 years. Thereafter, the effect of clarithromycin on all outcomes almost dissipated.

### Sensitivity analyses

When adding three comorbidities hyperlipidemia, renal diseases, and arrhythmias (Supplementary Table 2) and one confounding drug quinolone (Supplementary Table 3) in the original regression model, the results remained consistent. When considering amoxicillin users as controls (Supplementary Table 4), the results remained similar. We also found cardiovascular outcomes were not associated with clarithromycin indications (Supplementary Table 5) and dosing period of clarithromycin indications for acute respiratory tract infection and *Helicobacter pylori* infection (Supplementary Table 6).

## Discussion

In this nationwide cohort study using a single-payer national healthcare insurance database over a 10-year period of follow-up, we used propensity score matching and new user design to demonstrate that the adverse effect of clarithromycin use *vs.* non-use on the increased all-cause mortality, cardiovascular mortality, non-cardiovascular mortality, and cardiovascular morbidity (especially heart events) in patients with stable CHD occurred predominately during the first 3 years of follow-up, then almost dissipated afterward. We also found cardiac angiography without percutaneous transluminal coronary angioplasty, not percutaneous transluminal coronary angioplasty and cardiac electrophysiological study, seemed to predominately account for unstable heart conditions. Notably, we found no dose-response and dosing period relationships between clarithromycin use and cardiovascular outcomes, and no relationship between clarithromycin indications and cardiovascular outcomes.

As mentioned above, prospective, randomized-controlled trials (6–10) and observational studies (12–16) evaluated the efficacy and risk between macrolide antibiotics and cardiovascular events. The results were conflicting in those studies. For example, a randomized, double-blind study enrolled 148 patients with acute non-ST elevation myocardial infarction or unstable angina. These patients were treated with clarithromycin or placebo for 3 months, and it concluded that cardiovascular events would be reduced among patients administered clarithromycin (9). However, another prospective study (CLARICOR trial) evaluated the outcome of clarithromycin users with stable heart disease and reported poor outcome (7, 22). In the CLARICOR trial, short-term (2 weeks) use of clarithromycin among patients with stable CHD was related to a higher cardiovascular mortality rate after a 3-year follow-up (7). But the risk was reversed in another study after longer-term follow-up to 10 years (22). In the present study, we assessed cardiovascular outcomes from short-term to long-term in stable CHD patients after receiving clarithromycin. We found cardiovascular mortality, non-cardiovascular mortality, and cardiovascular morbidity mainly occurred in short-term follow-up (first 3 years after clarithromycin use). Consistent with our results, two previous studies (16, 22) also demonstrated the short-term adverse effect on all-cause, cause-specific mortality, and cardiovascular morbidity. Though the short-term and long-term outcomes looked paradoxical, there may exist depletion of the susceptible effect (46) in patients using clarithromycin. Patients susceptible to the adverse impact emerged first, and those remaining in the cohort were possibly more resistant to the side effect. The possible adverse effect of clarithromycin on the heart may be due to the side effect of macrolide antibiotics. It was thought that macrolide antimicrobials would delay the ventricular repolarization and *torsades de pointes* may occur after QT prolongation in patients with risk factors (47). However, it seemed to be contrast to our result that only cardiac angiography without percutaneous transluminal coronary angioplasty was significantly associated with increased risk of unstable heart conditions in clarithromycin users. However, we found percutaneous transluminal coronary angioplasty and cardiac electrophysiological study were not significantly associated with increased risk of unstable heart conditions, although limited events. These results were consistent to a study reporting no increased hazards for arrhythmias and acute myocardial infarction in multivariate analyses (45). Another study evaluated the safety of macrolide and fluoroquinolone, including clarithromycin, in patients with multidrug-resistant tuberculosis or non-tuberculous mycobacterial disease (48). They found that though QT prolongation occurred in some patients, no significant clinical events were observed. Nevertheless, when we use these antibiotics in CHD patients, we should consider co-morbidities in different populations to avoid adverse effects.

Notably, our results demonstrated no dose-response relationship between clarithromycin and cardiovascular outcomes. The depletion of the susceptible effect might account for this phenomenon. Moreover, patients diagnosed with infection but receiving less clarithromycin might also have more severe sepsis or drug-resistant bacteria infection. Hence, their antimicrobials were changed to much broader or newer generations of antibiotics. Severe sepsis itself resulted in higher mortality and also partly explained why a higher non-cardiovascular mortality rate occurred in these clarithromycin users. Our results also demonstrated no dosing period relationship between clarithromycin and cardiovascular outcomes. Another study (49) revealed that longer courses of clarithromycin were associated with more cardiovascular events in those with lower respiratory tract infection, but they separated the dosing periods into < 3 days, 3–6 days, 7 days, and > 7 days. From that study, we may not interpret that a prolonged course of clarithromycin was related to poor cardiovascular outcomes. Their finding might also reveal that the duration of antibiotics treatment represented the severity of the illness.

We further analyzed cardiovascular morbidities including heart, stroke, and PAOD events. We found the higher cardiovascular morbidity risk among clarithromycin users during the first 3 years was mainly from heart events, whereas clarithromycin use was not associated with increased risk for stroke and PAOD events during the first 3 years, at 3–6 years, or after 6 years of follow-up. Concerning cardiovascular morbidities, previous studies had conflicting results. A study found that clarithromycin was related to a reduced risk of arrhythmia and stroke (15); and a study showed clarithromycin was not associated with a higher rate of arrhythmia (50); but another study reported an increased risk of stroke (14). The CLARICOR trial with follow-up for 10 years demonstrated that the increased risk of cardiovascular mortality and stroke occurred mainly among patients not using statin (22). Furthermore, all-cause mortality, cardiovascular mortality, and cerebrovascular events among those statin users were not significantly different between clarithromycin and placebo groups during their 10-year follow-up period. These results suggested that clarithromycin and statin both contributed to the outcomes in the previous CLARICOR trial. The present study also considered statin a confounding drug and included it in the propensity score matching, which made no difference between clarithromycin users and non-users. Therefore, clarithromycin itself may account for these risks in our analyses.

By analyzing the NHI claims data with a highly representative sample, the present study has five strengths. First, recall bias of clarithromycin was avoided. Second, using the new-user design minimized the immortal bias and the potential residual effect of using clarithromycin before CHD inception date. Third, the follow-up of death and cardiovascular outcomes was complete, and the use of competing mortality minimized risk overestimation of cardiovascular morbidity. Fourth, use of propensity score matching minimized confounding effects. Fifth, consideration of medical services minimized detection bias. However, several potential limitations exist. First, the NHI claims data lack information on lifestyle (e.g., smoking, alcohol consumption, diet, and physical activity), body weight, blood pressure and sugar levels, and laboratory data, which may contribute to the risks of death and cardiovascular outcome. Second, the compliance of prescribed clarithromycin was not assessed in the administrative claims. Third, this study was not a prospective design. However, when we assumed the association between clarithromycin and cardiac harm existed, it was not reasonable to conduct a prospective study similar to the previous one. Besides, considering the overuse of antibiotics nowadays, it may be better to perform retrospective studies to evaluate the impact of clarithromycin in patients with CHD. Moreover, antimicrobials were chosen according to individual conditions, so it is not reasonable to randomly assign one patient with CHD diagnosed with infectious disease to receive clarithromycin and the other not when indicated. Fourth, the diagnosis of CHD was based on ICD-9-CM codes and misclassification is possible. However, this method has been used in prior NHIRD-based research (29, 30) and a previous study (51) also documented that the NHIRD appears to be a valid resource for population research in CVDs. Fifth, drug utilization data presented in DDDs provide an overall estimate of utilization and may not represent actual trends in use (44). Finally, as with any observational study, residual confounding by unmeasured factors that were different between users and non-users may still exist (31). However, the confounding effect of medical attention could be corrected for by inclusion of the number of medical visits in the propensity score matching and regression model (31).

## Conclusion

The nationwide retrospective cohort study documented that clarithromycin use was associated with increased risk for short-term cardiovascular morbidity (especially, heart events) and mortality without a dose-response relationship in patients with stable CHD. This association was not dose dependent and confounded by indications. Hence, patients with stable CHD while receiving clarithromycin should watch for these short-term potential risks.

## Data availability statement

The datasets utilized in this study cannot be made available in a public repository due to the “Personal Information Protection Act” executed by Taiwan’s government, starting from 2012. Requests to access these datasets should be directed to the corresponding author.

## Ethics statement

The studies involving human participants were reviewed and approved by the Institutional Review Board of the Dalin Tzu Chi Hospital (B10702014 and B11101016). Written informed consent for participation was not provided by the participants’ legal guardians/next of kin because: The NHIRD encrypts patients’ personal information to protect privacy and provides researchers with anonymous identification numbers associated with relevant claims information, including sex, date of birth, medical services received, and prescriptions. Therefore, patient consent is not required to access the NHIRD. This study was approved by the Institutional Review Board (IRB) of Dalin Tzu Chi Hospital; the IRB also waived the consent requirement.

## Author contributions

B-HY, Ye-CC, and Yi-CC contributed to study design. Ye-CC contributed to administrative support. B-HY, W-YC, and Yi-CC contributed to collection and data assembly. B-HY, Ye-CC, Y-DL, W-YC, and Yi-CC contributed to analysis and interpretation of data. Ye-CC and Yi-CC contributed to manuscript writing. Yi-CC was responsible for the overall content as the guarantor. All authors involved in revising the manuscript for important intellectual content and approved the final revision to be published, contributed to the article and approved the submitted version.
