# Supplementary material for: No dose-response relationship of clarithromycin utilization on cardiovascular outcomes in patients with stable coronary heart disease: Analysis of Taiwan’s national health insurance claims data
Source: Front Cardiovasc Med. 2022 Oct 26;9:1018194. doi: 10.3389/fcvm.2022.1018194 (PMC9645004; doi:10.3389/fcvm.2022.1018194)
Supplement: Supplementary file 1 [file Data_Sheet_1.pdf]

Supplemental Table 1. Association of clarithromycin with unstable heart conditions[†]

	Clarithromycin users (n=9,631)	Nonusers (n=9,631)	*p*-value	Adjusted HR[#] (95% CI)	*p*-value
Percutaneous transluminal coronary angioplasty				1.19 (0.88-1.61)	0.25
Mean follow-up (±SD)	4.0±2.9	4.1±2.9			
Event (n, %)	95 (1.0)	76 (0.8)	0.25		
Cumulative incidence (%, 95% CI)	1.9 (1.5-2.4)	1.6 (1.2-2.0)	0.24		
Only cardiac angiography without percutaneous transluminal coronary angioplasty				1.32 (1.08-1.61)	0.007
Mean follow-up (±SD)	4.0±2.9	4.1±2.9			
Event (n, %)	227 (2.4)	164 (1.8)	0.006		
Cumulative incidence (%, 95% CI)	4.1 (3.5-4.8)	3.2 (2.6-3.8)	0.005		
Cardiac electrophysiological study				2.01 (0.20-20.29)	0.55
Mean follow-up (±SD)	4.0±2.9	4.1±2.9			
Event (n, %)	2 (0.02)	1 (0.01)	0.59		
Cumulative incidence (%, 95% CI)	0.03 (0.01-0.09)	0.02 (0.00-0.09)	0.59		

Abbreviations: SD, standard deviation; HR, hazard ratio; CI, confidence interval.

[†]defined as experience of any of three cardiac interventional procedures, whichever came first.

[#]Adjusted for all covariates (age per year, sex, comorbidity, number of medical visits, and confounding drugs) and competing mortality.

Supplemental Table 2. Sensitivity analysis 1: adding three comorbidities hyperlipidemia, renal diseases, and arrhythmias into the original regression model listed in Table 2

	Overall mortality aHR* (95% CI)	Cardiovascular mortality aHR* (95% CI)	Cardiovascular morbidity aHR# (95% CI)
Nonusers (n=9631)	1 (Reference)	1 (Reference)	1 (Reference)
Clarithromycin users (n=9631)	1.41 (1.27-1.56)	1.33 (1.07-1.64)	1.11 (0.96-1.29)

Abbreviations: HR, hazard ratio; CI, confidence interval.

*Adjusted for all covariates (age per year, sex, comorbidity, Charlson comorbidity index, number of medical visits, and drugs use) listed in Table 1.

#Adjusted for all covariates (age per year, sex, comorbidity, Charlson comorbidity index, number of medical visits, and drugs use) listed in Table 1 and competing mortality.

Supplemental Table 3. Sensitivity analysis 2: adding one confounding drug quinolone antibiotic into the original regression model listed in Table 2

	Overall mortality aHR* (95% CI)	Cardiovascular mortality aHR* (95% CI)	Cardiovascular morbidity aHR# (95% CI)
Nonusers (n=9631)	1 (Reference)	1 (Reference)	1 (Reference)
Clarithromycin users (n=9631)	1.43 (1.29-1.58)	1.36 (1.10-1.68)	1.11 (0.96-1.29)

Abbreviations: HR, hazard ratio; CI, confidence interval.

*Adjusted for all covariates (age per year, sex, comorbidity, Charlson comorbidity index, number of medical visits, and drugs use) listed in Table 1.

#Adjusted for all covariates (age per year, sex, comorbidity, Charlson comorbidity index, number of medical visits, and drugs use) listed in Table 1 and competing mortality.

Supplemental Table 4. Sensitivity analysis 3: study outcomes by clarithromycin use *vs.* amoxicillin use (as control group)

Take amoxicillin as control group	Overall mortality aHR* (95% CI)	Cardiovascular mortality aHR* (95% CI)	Cardiovascular morbidity aHR# (95% CI)
**Model 1:** consider the same covariates as those listed in Table 1 and propensity score matching			
Amoxicillin users (n=7445)	1 (Reference)	1 (Reference)	1 (Reference)
Clarithromycin users (n=7445)	1.47 (1.27-1.70)	1.44 (1.05-1.99)	0.50 (0.44-0.57)
**Model 2:** add new four covariates: three comorbidities (hyperlipidemia, renal diseases, and arrhythmias) and one confounding drug (quinolone) in addition to the original covariates listed in Table 1 and consider propensity score matching			
Amoxicillin users (n=5851)	1 (Reference)	1 (Reference)	1 (Reference)
Clarithromycin users (n=5851)	1.56 (1.30-1.87)	1.54 (1.01-2.34)	0.52 (0.45-0.61)

Abbreviations: HR, hazard ratio; CI, confidence interval.

*Adjusted for all covariates (age per year, sex, comorbidity, Charlson comorbidity index, number of medical visits, and drugs use listed in Table 1.

#Adjusted for all covariates (age per year, sex, comorbidity, Charlson comorbidity index, number of medical visits, and drugs use listed in Table 1 and competing mortality.

Supplemental Table 5. Subgroup analysis of cardiovascular outcomes by clarithromycin indications

	Clarithromycin users (n=9631)			Nonusers (n=9631)			Adjusted hazard ratio (95% CI)			
	n	CV morbidity events	CV mortality events	n	CV morbidity events	CV mortality events	CV morbidity*	*p-value*	CV mortality#	*p-value*
Acute respiratory infection										
No	2241	127	45	2412	254	38	1.14 (0.85-1.50)	0.36	1.55 (1.00-2.40)	0.05
Yes	7390	349	145	7219	613	124	1.11 (0.94-1.32)	0.22	1.31 (1.03-1.66)	0.03
Genitourinary tract infection										
No	8008	423	170	8082	765	138	1.14 (0.97-1.32)	0.10	1.42 (1.13-1.78)	0.002
Yes	1623	53	20	1549	102	24	0.98 (0.65-1.50)	0.93	0.93 (0.50-1.73)	0.82
Helicobacter pylori infection										
No	9616	9630	9616	9624	9630	9624	1.12 (0.97-1.29)	0.13	1.35 (1.09-1.66)	0.006
Yes	15	1	15	7	1	7	Not converged		Not converged	
Tuberculosis infection										
No	9561	473	185	9580	863	156	1.11 (0.96-1.29)	0.15	1.38 (1.11-1.70)	0.004
Yes	70	3	5	51	4	6	3.33 (0.10-108.38)	0.50	1.01 (0.19-5.49)	0.99
Non-tuberculosis										

infection										
No	9625	476	190	9631	867	162	1.12 (0.97-1.29)	0.14	1.35 (1.09-1.67)	0.005
Yes	6	0	0	0	0	0	-	-	--	-

*Adjusted for all covariates, including age year, sex, comorbidity (diabetes, hypertension, COPD, acute respiratory infection, genitourinary tract infection, *Helicobacter pylori* infection, mycobacterial [tuberculosis and non-tuberculosis] infection), number of medical visits, and confounding drugs, and competing mortality, minus the covariate on which stratified.

#Adjusted for all covariates, including age year, sex, comorbidity (diabetes, hypertension, COPD, acute respiratory infection, genitourinary tract infection, *Helicobacter pylori* infection, mycobacterial [tuberculosis and non-tuberculosis] infection), number of medical visits, and confounding drugs, minus the covariate on which stratified.

Supplemental Table 6. Subgroup analysis of cardiovascular outcomes by dosing period of clarithromycin indications for acute respiratory tract infection and *Helicobacter pylori* infection

Clarithromycin prescription	**Cardiovascular mortality aHR* (95% CI)**	**Cardiovascular morbidity aHR# (95% CI)**
None (n=9631)	1 (Reference)	1 (Reference)
≦7 days (n=6359)		
Acute respiratory tract infection		
No (n=1614)	2.46 (1.43-4.23)	1.25 (0.90-1.74)
Yes (n=4745)	0.63 (0.21-1.88)	0.74 (0.37-1.51)
Helicobacter pylori infection		
No (n=6439)	1.60 (1.23-2.08)	1.15 (0.96-1.37)
Yes (n=10)	Not converged	Not converged
8-14 days (n=1987)		
Acute respiratory tract infection		
No (n=421)	0.63 (0.21-1.88)	0.74 (0.37-1.51)
Yes (n=1566)	1.18 (0.70-2.00)	1.08 (0.74-1.59)
Helicobacter pylori infection		
No (n=1986)	1.00 (0.63-1.58)	0.97 (0.70-1.35)
Yes (n=1)	Not converged	Not converged
≧15 days (n=1285)		
Acute respiratory tract infection		
No (n=206)	0.31 (0.02-4.28)	0.95 (0.39-2.33)
Yes (n=1079)	0.90 (0.47-1.72)	1.22 (0.78-1.90)

Helicobacter pylori infection		
No (n=1281)	0.80 (0.44-1.47)	1.18 (0.79-1.75)
Yes (n=4)	Not converged	Not converged

Abbreviations: SD, standard deviation; aHR, adjusted hazard ratio; CI, confidence interval.

*Adjusted for all covariates (age per year, sex, comorbidity (diabetes, hypertension, chronic obstructive pulmonary disease, acute respiratory tract infection, and H. pylori infection), number of medical visits, and confounding drugs), minus the covariate on which stratified. #Adjusted for all covariates (age per year, sex, comorbidity (diabetes, hypertension, chronic obstructive pulmonary disease, acute respiratory tract infection, and H. pylori infection), number of medical visits, and confounding drugs), minus the covariate on which stratified, and competing mortality.
